# Mechanical Properties of Dental Composites Modified with Liquid Rubber and Their Effect on Stress Distribution in Fillings

**DOI:** 10.3390/ma18245664

**Published:** 2025-12-17

**Authors:** Monika Sowa, Leszek Borkowski, Krzysztof Pałka

**Affiliations:** 1 Faculty of Mechanical Engineering, Lublin University of Technology, Nadbystrzycka St. 36, 20-618 Lublin, Poland; d577@pollub.edu.pl; 2 Department of Biochemistry and Biotechnology, Medical University of Lublin, Chodzki St. 1, 20-093 Lublin, Poland; leszek.borkowski@umlub.edu.pl

**Keywords:** dental composite, liquid rubber, finite element method, mechanical properties

## Abstract

Dental composites are commonly used for the restoration of hard tooth tissues, but their low fracture toughness may limit their lifespan. In this study, the effect of liquid rubber modification on the mechanical properties and fracture mechanisms of two types of dental composites, flow and classic, was evaluated. The study used experimental composites containing a mixture of dimethacrylate resins: BisGMA (20% by weight), BisEMA (30% by weight), UDMA (30% by weight), and TEGDMA (20% by weight). Composites were reinforced with Al-Ba-B-Si glass, Ba-Al-B-F-Si glass with particle sizes of 0.7 and 2 μm respectively, as well as pyrogenic silica (20 nm). The inorganic phase was introduced in an amount of 50% vol. for flow material and 80% vol. for classic composite. As a modifier, Hypro 2000X168LC VTB liquid rubber (Huntsman International LLC, USA) was used in an amount of 5% by weight relative to the matrix. The flexural strength, Young’s modulus, and fracture toughness were evaluated. Numerical FEM analysis allowed for the evaluation of stress distribution in the filling area. The results confirmed that the modification of composites with liquid rubber contributes to an increase in fracture toughness. For the flow-type material, the fracture toughness increased from 1.04 to 1.13 MPa·m^1/2^. At the same time, a decrease in flexural strength from 71.90 MPa to 61.48 MPa and in Young’s modulus from 2.98 GPa to 2.53 GPa. In the case of the classical composite, the modification with liquid rubber also improved the resistance to fracture, increasing it from 1.97 to 2.18 MPa·m^1/2^ while the flexural strength decreased from 102.30 MPa to 90.96 MPa, and the modulus dropped from 7.33 GPa to 6.16 GPa. FEA analysis confirmed that modified composites exhibit a more favorable stress distribution with lower tensile stress levels (approximately 20 MPa in contrast to 25 MPa for the classic composite). Mechanisms of fracture and strengthening were also identified. The main fracture mechanism was intermolecular cracking with crack deflections. Modification with liquid rubber resulted in the formation of elastic bridges and plastic shear zones at the front of the crack.

## 1. Introduction

Modern civilization puts growing focus on protecting health and improving quality of life, including oral health. Prevention, early diagnosis, and effective treatment of dental and gum diseases are not only signs of medical progress but also an important part of modern healthcare. Dental composites have become the dominant material in modern conservative dentistry, combining aesthetics with functionality in the restoration of tooth tissues [1]. Their popularity results from their excellent application properties, which make them widely used in the treatment of carious lesions, ensuring aesthetic reconstruction of teeth damaged by disease [2]. However, dental composites have many imperfections, including fracture toughness, which is one of the major factors in the development of new dental composites. It is one of the key challenges in the development of new dental composites, as it causes cracking, and negatively affects the marginal integrity of the restoration [3]. resulting in reduced durability [4,5]. Under dynamic loads, such as chewing forces, emerging stresses accelerate filling degradation, leading to premature wear and the need for replacement.

Particular attention is paid to occlusal stresses, which act at specific points of contact between the tooth and the filling. It appears especially in areas of local stress concentrations (occlusal contact, presence of filling material with properties different from those of tooth tissue), which can promote the initiation of microcracks and the propagation of damage to the filling adjacent to the tooth tissue. This is particularly important when mechanical and thermal loads are combined, when maximum reduced stresses can reach values of almost 670 MPa [6]. Therefore, it is very important to manufacture composite materials that enable a uniform stress distribution to reduce the negative effects of stress accumulation.

Contemporary research focuses on finding strategies to developing modern composite materials with improved fracture toughness and flexural strength, thus increasing the stability and durability of dental fillings [7,8]. One strategy is to modify resins with an elastic phase, known as rubber reinforcement [9]. Improving the fracture toughness of methacrylate resins using low molecular weight liquid rubbers has been the subject of numerous studies [10,11]. Many researchers [10,12] have also noted that modifying polymers with liquid rubber improves their mechanical properties, including flexural strength and fracture toughness. The modification of resins with liquid rubber is well described in the context of their use in polymer systems [10,11]. However, to date, there has been little research on its use in dimethacrylate resins, which are the basic matrix of dental composites. The literature indicates that the introduction of liquid rubber improves mechanical properties of many polymeric materials, suggesting the possibility of similar effects in those based on dimethacrylates [13,14]. The work [15] described liquid rubber for modification of the dimethacrylate matrix used in dental composites. In the experiment, different amounts of liquid rubber were added to two commercial dimethacrylate resin mixtures to test the selected mechanical properties. It was found that with the appropriate content of liquid rubber (5% by weight relative to the matrix), a significant increase in fracture toughness was achieved.

The aim of the study is to modify an experimental dental composite with liquid rubber and to evaluate its influence on mechanical properties, stress distribution in fillings, and fracture mechanisms. The null hypothesis assumes that the addition of liquid rubber will positively affect the stress distribution of the material used for dental fillings, as well as the initiation and propagation of cracks, contributing to improved fracture toughness of the composite material and increasing its durability under functional loads.

## 2. Materials and Methods

### 2.1. Materials

The material used in the study consisted of two experimental composites: a flow-type composite and a classic composite, manufactured for research purposes by the ARKONA Dental Pharmacology Laboratory (Nasutów, Poland). The composites developed in this study were experimental materials intended solely for research purposes rather than clinical application. The primary aim was to evaluate how liquid rubber modification influences their properties and mechanical behavior.

Preliminary studies have shown that the mechanically optimal matrix composition is as follows:

- BisGMA (20% by weight)
- BisEMA (30% by weight),
- UDMA (30% by weight),
- and TEGDMA (20% by weight).
- photoinitiator (camphorquinone) 1% by weight of,
- co-initiator (2-dimethylaminoethyl methacrylate, DMAEMA),
- and an inhibitor (butylated hydroxytoluene, BHT).

All resins and additives were purchased from Sigma-Aldrich Chemicals (Munich, Germany). The reinforcement in both cases had the same composition, which was a mixture of two types of glass:

- Al-Ba-B-Si glass, with an average particle size of 0.7 μm,
- Ba-Al-B-F-Si glass, with an average particle size 0.2 μm),
- pyrogenic silica (20 nm).

All the reinforcement materials were also purchased from Sigma-Aldrich Chemicals. (Munich, Germany).

The methacrylate resins employed in this study are commonly used in a wide range of dental composites. Likewise, the selected reinforcement material and particle size are characteristic of micro-hybrid composites. These components were chosen for their versatility, enabling a systematic evaluation of the effects of liquid rubber modification.

The amount of reinforcement was 50% and 80% vol., respectively, in the flow-type composite and the classic one. As a modifier, liquid rubber Hypro 2000X168LC VTB (Huntsman International LLC, Woodlands, TX, USA) was used, added in an amount of 5% by weight relative to the matrix. Unmodified composites were used as reference material. The test samples were designated according to Table 1.

### 2.2. Mechanical Properties

#### 2.2.1. Fracture Toughness

The fracture toughness *K_IC_* was evaluated according to ASTM E 399-20 standard [16], using notched samples. The tests were carried out on an Autograph AG-X Plus testing machine (Shimadzu, Kioto, Japan), using a force transducer with a range of 1 kN and a crosshead rate of 0.5 mm/min. The *K_IC_ *[MPa m^1/2^] was determined according to the formula (all variables are explained below):

(1) $$K_{IC}=\frac{\sqrt[{3}]{\frac{A}{W}}\left[1.99-\frac{A}{W}\left(1-\frac{A}{W}\right)\left(2.15-3.93\frac{A}{W}+2.7{\left(\frac{A}{W}\right)}^{2}\right)\right]PL}{2(1+2\frac{A}{W}){\left(1-\frac{A}{W}\right)}^{\frac{2}{3}}{BW}^{\frac{3}{2}}}$$

The samples for the test were prepared according to the ASTM standard [16]. The transverse dimensions of the sample met the condition 1 *W*/*B* 4 where *W* is the height and *B* is the width of the sample. Additionally, the distance between the supports was *L* = 4 *W*. The depth of the notch *a* measured after breaking the sample met the condition 0.45 *W* < *a* < 0.55 *W*. Considering the above, the dimensions of the samples were 15 mm × 2.2 mm × 2.2 mm (respectively, *L*_specimen_ × *W* × *B*), the distance between supports *L* = 8.8 mm and the notch angle of 80°.

The samples were polymerized using a 1350 mW/cm^2^ LED lamp for 20 s at each irradiated area, with a Mylar-type foil applied to prevent surface oxidation. The irradiation of the sample was performed in overlapping zones as follows: the central region was cured first, after which the optical fiber was moved to the left, partially overlapping the previously cured area. Following curing of this second region, the fiber was shifted to the right of the central zone, again partially overlapping this region. This procedure was repeated sequentially on both sides of the central area until the entire specimen was fully polymerized.

#### 2.2.2. Flexural Strength

The flexural strength *σ_f_* [MPa] was determined in a three-point bending test according to ISO 4049:2019 [17]. Mechanical testing was carried out with an Autograph AG-X Plus (Shimadzu, Kioto, Japan) at a speed of 0.5 mm/min using the 1 kN load cell with accuracy class 0.1. The flexural strength was calculated using the following formula:

(2) $$\sigma_{F}=\frac{3PL}{2{BW}^{2}}$$

where: *P* is the maximum force at specimen fracture. The samples for the flexural strength test were prepared according to ISO 4049 [17], with the following dimensions: 25 mm × 2 mm × 2 mm (length × height × width), with a support span *L* of 20 mm. The same lamp was used for polymerization of the samples as was used in the fracture toughness test, the polymerization method was also the same.

#### 2.2.3. Young’s Modulus

Young’s modulus *E* [GPa] was calculated based on EN ISO 178:2019 [18], using data from the bending test according to the formula:

(3) $$E=\frac{\sigma_{f2}-\sigma_{f1}}{\varepsilon_{f2}-\varepsilon_{f1}}$$

where: *ε_f_*_1_ = 0.0005, *ε_f_*_2_ = 0.0025, while *σ_f_*_1_ and *σ_f_*_2_ are stresses calculated for strains *ε_f_*_1_ and *ε_f_*_2_.

#### 2.2.4. The Poisson’s Ratio

The Poisson ratio was experimentally determined using the digital image correlation (DIC) method with the Aramis 12M system (GOM, Braunschweig, Germany). Cylindrical samples measuring 12 mm × 4.7 mm (height and diameter, respectively) were coated with a contrasting substance, which allowed tracking of displacements during compression testing carried out at a speed of 1 mm/s using Autograph AG-X Plus testing machine (Shimadzu, Kioto, Japan).

Based on the recorded images, the displacement field was reconstructed. Virtual extensometers were created in selected measurement areas, allowing for displacement determination along both the perpendicular and parallel directions to compression. On this basis, local longitudinal and transverse strains were calculated, and Poisson’s ratio (*ν*) was determined according to the well-known formula:

(4) $$\upsilon =-\frac{\varepsilon_{transverse}}{\varepsilon_{longitudinal}}$$

### 2.3. Finite Element Analysis

Table 2 summarizes the mechanical properties of the tooth tissues used for the numerical analysis, based on work [19].

**Figure 1:**
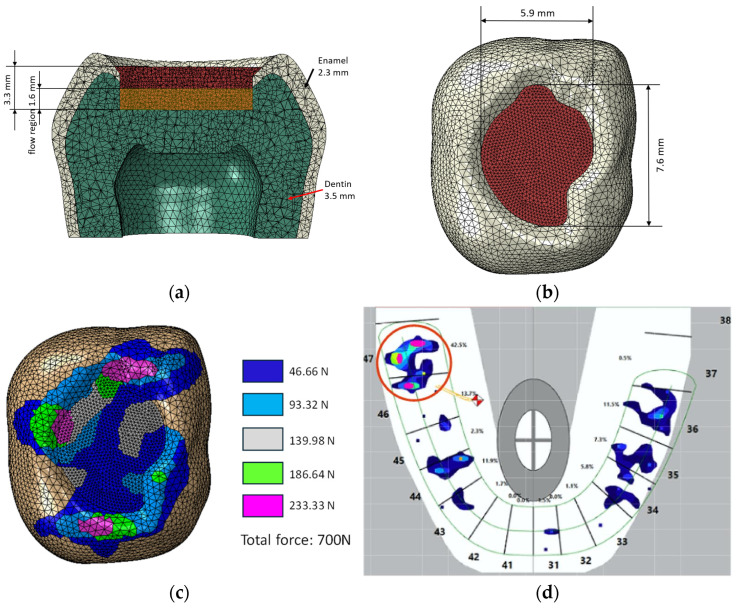
(**a**) MES model with composite layers in filling, (**b**) dimensions of the filling, (**c**) force application areas, (**d**) distribution of forces on molar tooth 47 (in red circle) according to results from the T-scan Novum device, color scale is proportional to that of (**c**).

A cross-section of the model is shown in Figure 1a and dimensions of the filling in Figure 1b. In the tooth model, a two-layer filling was used, consisting of a flow-type filling in the dentin area and a classic composite in the enamel area. Two sets of composites: reference materials and composites modified with liquid rubber were used in the simulation. The finite element model consisted of 273,315 nodes and 187,126 C3D10HS elements. Meshing was performed with a global element size of 0.6 mm, with curvature control enabled to ensure automatic refinement in regions of high curvature, such as the fillings and enamel–cavity interfaces. The minimum element size was 0.18 mm. The bottom surface of the model was fixed, constraining all translational degrees of freedom (UX = UY = UZ = 0), while the remaining regions were left unconstrained, allowing full deformation under applied loads. The adhesive layer between the filling material and the hard-dental tissue has been taken into consideration in the model; for that purpose, the contact interactions of the surface-based cohesive behavior type.

The purpose of the numerical simulations was to compare stress distribution on the surface of the restoration. Consequently, no convergence analysis was conducted for this study. A total load of 700 N was applied through coupling interactions (Figure 1c), and the points of force application corresponded to the actual case of a molar tooth, for which the distribution of the occlusal forces was assessed using a T-scan Novum (Tekskan, Norwood, MA, USA) (Figure 1c). Although the loads depicted in Figure 1d are not perfectly balanced, this model was accepted to simulate an extreme and highly unfavorable loading condition.

The ABAQUS/CAE 2025 was used for numerical analysis. The numerical calculation process used included nonlinear geometric analysis using the Newton-Raphson method [20]. For all areas of the model: enamel, dentin and filling area, a material model with linear-elastic characteristics was defined. This model was considered appropriate because the addition of 5% by weight of liquid rubber does not induce substantial changes in the mechanical response of the composites, making a linear analysis reasonable at this stage of the study.

The simulation yielded the following results: the distributions of the maximum principal stresses (S. Max. Principal) and reduced stresses according to the Huber-Mises-Hencky hypothesis (H-M-H). They allowed the evaluation of the stress state in the filling area. It was also possible to determine potential critical points within the filling and compare the behavior of modified and unmodified composites.

### 2.4. Statistical Evaluation

Statistical analysis was performed to determine statistically significant differences between the study groups. Statistical analysis was performed on 40 results collected in 4 groups/populations, each containing 10 replicates (*n* = 10). The D’Agostino & Pearson normality test was used to test for the normal distribution within each group. Because a normal distribution was confirmed, comparisons between groups were made using one-way analysis of variance (ANOVA) and Tukey’s post hoc test. All statistical analyses were conducted using GraphPad Prism (Version 7.04, San Diego, CA, USA). The results of the statistical analysis were indicated as follows: * *p* < 0.05.

## 3. Results

### 3.1. Results of Mechanical Tests

#### 3.1.1. The Fracture Toughness

**Figure 2:**
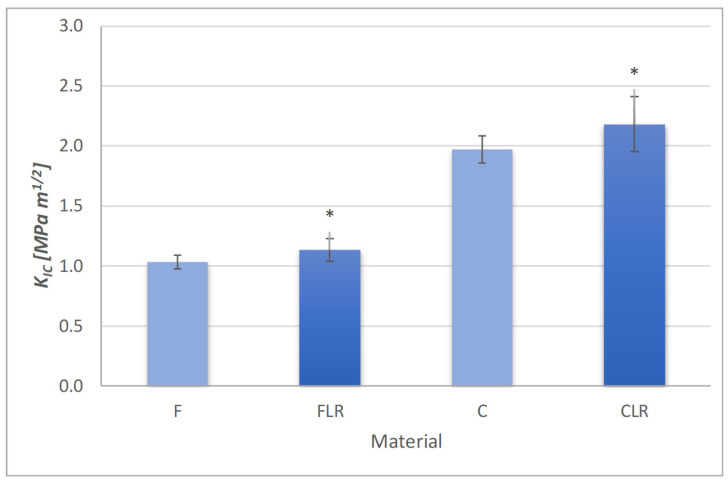
Fracture toughness of experimental dental composites. Asterisks indicate statistically significant differences for FLR and CLR material in relation to their reference materials (F and C, respectively).

The results of the fracture toughness test are presented in Figure 2. The modification with liquid rubber increased the fracture toughness for both the flow and classic composites. For the flow material, the fracture toughness was 1.04 MPa·m^1/2^, while after modification with liquid rubber it increased to 1.13 MPa·m^1/2^. In the case of the classic material, this value was 1.97 MPa·m^1/2^ and after applying liquid rubber it increased to 2.18 MPa·m^1/2^.

The higher amount of reinforcement in the case of the classic composite allowed a higher value for fracture toughness. Modification with liquid rubber had a positive effect on fracture toughness in both types of composites. In both cases, a statistically significant increase in fracture toughness after modification with liquid rubber was observed.

#### 3.1.2. Results of the Bending Test

**Figure 3:**
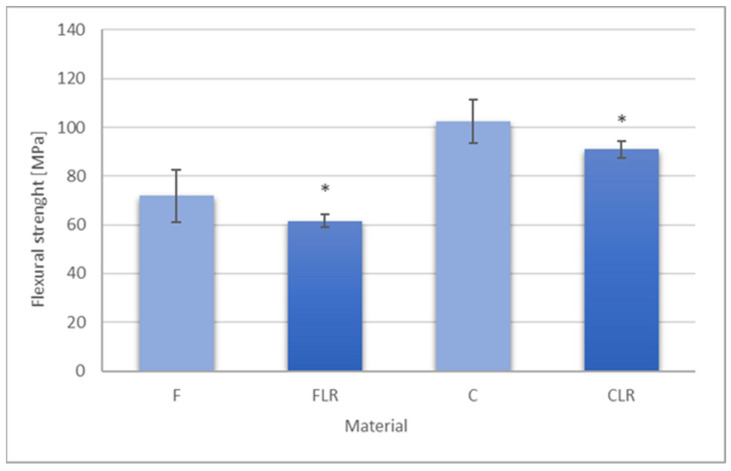
Flexural strength of experimental dental composites. Asterisks indicate statistically significant differences for FLR and CLR material in relation to their reference materials (F and C, respectively).

The results of the flexural strength for the tested composites are presented in Figure 3. The use of the modifier resulted in a statistically significant reduction in the value of flexural strength. For the flow type composite, the flexural strength decreased from 71.90 MPa to 61.48 MPa. These values categorize flow-type materials into materials as Type 2, Class 2, and group 1 materials according to EN ISO 4049:2019 [17], for which the minimum flexural strength requirement is 50 MPa. These materials can be used in areas where there are no occlusal forces (e.g., dentin). For the classic composite modified with liquid rubber, the flexural strength decreased from 102.30 MPa to 90.96 MPa, and it is still above the minimum requirements of EN ISO 4049 (80 MPa) type 1, class 2, group 1.

The above allows both materials to be used in clinical settings.

#### 3.1.3. The Young Modulus

**Figure 4:**
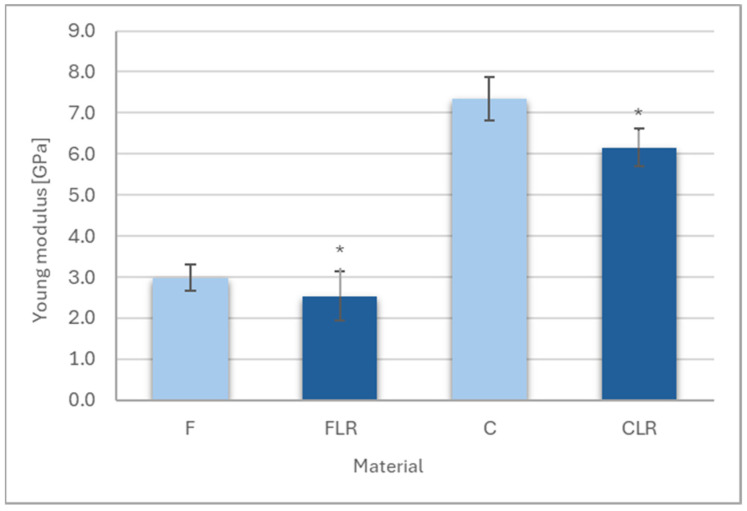
Young’s modulus of experimental dental composites. Asterisks indicate statistically significant differences between unmodified and rubber-modified materials within both the flowable and conventional composite groups.

The results of Young’s modulus measurements are shown in Figure 4. A statistically significant decrease in Young’s modulus values was observed for materials with liquid rubber content for both flow and classic material. Young’s modulus for F and C materials was 2.98 GPa and 7.33 GPa, respectively, while after modification with liquid rubber for FLR material, Young’s modulus was 2.53 GPa and for CLR it was 6.15 GPa.

### 3.2. Results of the Finite Element Analysis

Finite element analysis (FEA) was performed by simulating the occlusal forces on a tooth model created in Solid Edge ver. 2025.2410 software. The material properties obtained for the experimental composites are listed in Table 3.

**Figure 5:**
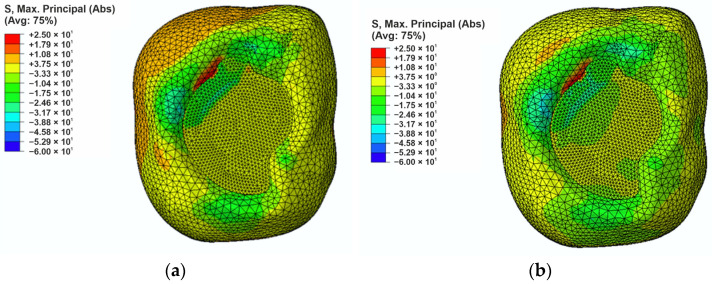
The maximum principal stress distribution in the filling region: (**a**) for the reference composite, and (**b**) for composite modified with liquid rubber.

Finite element analysis was performed using ABAQUS/CAE software which enabled the simulation of stress distribution in the tooth model. In the study two analyses were performed: the first involved filling with reference (unmodified) composites (Figure 5a), while the second included composites modified with liquid rubber (Figure 5b). As a result, the distributions of the maximum principal stresses (S. Max. Principal) and reduced stresses according to the Huber-Mises-Hencky hypothesis (H-M-H) were presented.

In the case of the reference filling, the maximum principal stress of approximately +25 MPa was observed at the interface between the filling and the enamel, while the lowest stress occurred at the apex of the cusp and its value was −53 MPa concentrated at the apex of the enamel. For the liquid rubber modified composite (Figure 5b) the highest principal stress, which was approximately +20 MPa, was observed on the enamel surface adjacent to the filling. The analysis showed that modification of composites with liquid rubber allows for a reduction in principal stresses of approximately 5 MPa, especially in areas exposed to direct occlusal forces. As a result of the modification of the filling material, the maximum principal stresses accumulated mainly in the enamel areas and not in the filling. In the case of the reference composite, a significant part of the minimum principal stresses was transferred to the filling, and the value of these stresses was 8 MPa in the filling-enamel contact area. This is an unfavorable phenomenon that can lead to higher stress and, consequently, to easier cracking and degradation of the filling, which negatively affects its durability. The composite modified with liquid rubber allows for a more favorable distribution of principal stresses in the filling (even a 50% reduction), which decreases the risk of crack propagation and mechanical damage.

**Figure 6:**
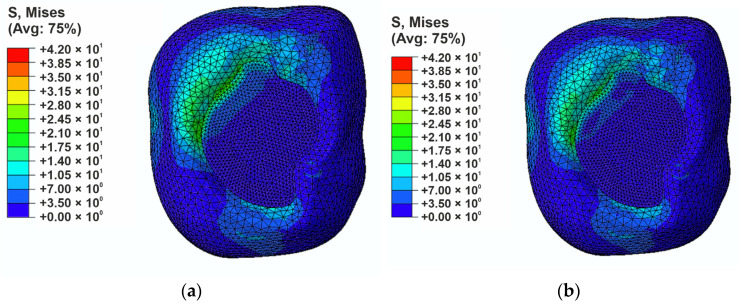
H-M-H stresses distribution in the filling region: (**a**) for the reference composite (**b**) for modified composite.

To comprehensively assess the stress state in the filling region, a stress analysis was performed according to the Huber–Mises–Hencky hypothesis (Figure 6). The maximum reduced stress values are comparable in both models (approximately 41 MPa). Differences are visible in the stress distribution, especially in the filling region and in the upper part of the area where a maximum force is applied. In the reference model (Figure 6a), the areas of increased stress are more concentrated and cover a wider range at the border between the filling and the enamel. This may lead to a higher risk of marginal gap appearance. In the case of the liquid rubber-modified composite (Figure 6b), the stresses show a more even distribution and are slightly more dispersed.

The results of both analyses confirmed that the modification of composites contributes to a reduction in local stress concentrations.

### 3.3. Microscopic Analysis of Fractures

The fracture mechanism of composite materials is significant in their microstructure, not only in the type and distribution of the reinforcement particles, but also on the phases that modify the matrix [21]. Structural differences between the composites tested affect the way the energy of the fracture is dispersed, which translates into their mechanical properties, especially fracture toughness. To identify the dominant fracture mechanisms, a microscopic analysis of the fracture surfaces of samples subjected to fracture toughness was performed using the Nova NanoSEM 450 microscopy (FEI, Eindhoven, The Netherlands. Observations were performed on side of samples that had fractured but still retained their integrity (side view of the crack) and also on the fracture surface

**Figure 7:**
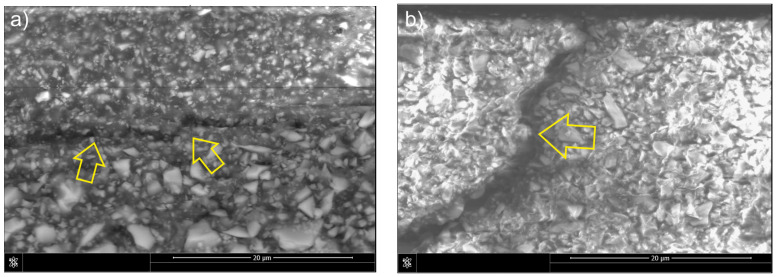
Mechanisms of fracture in composite F: (**a**) crack deflection, side view of the crack; (**b**) crack deflection on particle, fracture surface view of the sample. Arrows indicate the features present.

In the case of the F-type composite, classic fracture energy dissipation mechanisms were observed, including crack bifurcation and deflections on the reinforcement particles (Figure 7a). The cracks changed their propagation direction, which indicates an intense interaction with the filler particles. Figure 7b also shows deflections that may have been caused by local shear stresses in the matrix. The absence of elastic phases may have caused the crack to propagate relatively easily along the path of the lowest fracture energy, as confirmed by the fracture toughness value.

**Figure 8:**
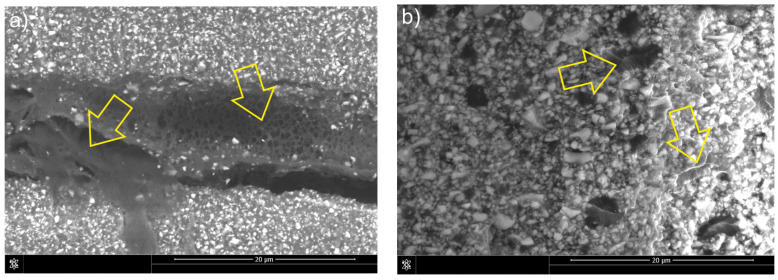
Mechanisms of fracture in FLR composite: (**a**) formation of elastic bridges, side view of the crack; (**b**) formation of elastic bridges, fracture surface view of the sample. Arrows indicate the features present.

The addition of liquid rubber in the case of the FLR composite resulted in a significant change in fracture mechanisms. Elastic bridges connecting the fracture edges were observed on the crack surface (Figure 8a), which absorbed energy and hindered further fracture propagation. The presence of the rubber phase allows for the formation of local elastic-plastic deformations and thus effective stress dissipation. It is also possible for a plastic deformation zone to form in front of the tip of the crack. In addition, blockage of the crack front by reinforcement particles and microdeformations near the rubber phase is visible (Figure 8b). This combination of mechanisms indicates a synergistic effect of both the liquid rubber and the reinforcement, which significantly increases the fracture toughness of the composite.

**Figure 9:**
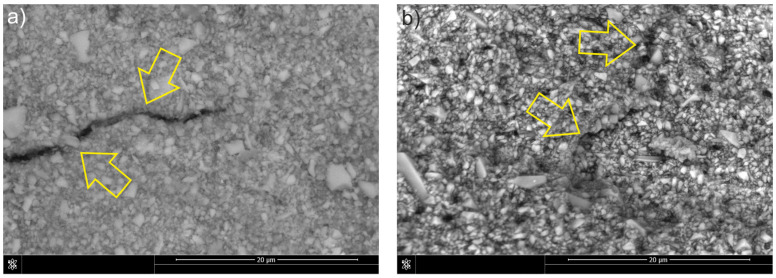
Mechanisms of fracture in C composite: (**a**) crack deflection and particle bypassing, side view of the crack; (**b**) microcrack formation, fracture surface view of the sample. Arrows indicate the features present.

The dominant fracture mechanism of the unmodified classic composite (C) was the interparticle one (Figure 9a), similar to that of the composite (F). However, due to the higher amount of reinforcement, fewer deflections and particle bypass incidents were observed during fracture propagation. The presence of numerous filler particles caused local changes in the direction of fracture propagation and microcrack formation (Figure 9b), which contributed to the dissipation of fracture energy and limited the development of the main fracture. These phenomena are characteristic of a brittle failure mechanism with a local bifurcation effect and deflection of the fracture.

**Figure 10:**
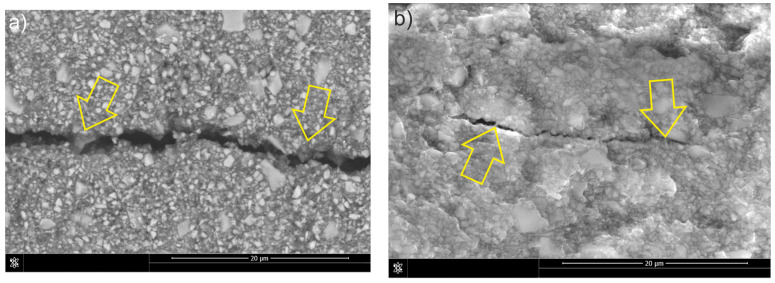
Mechanisms of fracture in CLR composite: (**a**) bypassing the particles and formation of elastic bridges, side view of the crack; (**b**) formation of elastic bridges in microcrack, fracture surface view of the sample. Arrows indicate the features present.

The introduction of liquid rubber into the classic composite (CLR) significantly affected the mechanics of the fracture (Figure 10a). Interparticle cracking is still present; however, the reduced amount of resin (compared to flow composites) resulted in a reduction in the number of elastic bridges (Figure 10b). The presence of dissolved rubber in the matrix may promote the initiation of plastic shearing and cause significant deflection of the crack tip, which in turn dissipates the fracture energy. The synergistic effects of cavitation localized at the rubber-resin interface and plastic shearing in the resin are presumably responsible for the deformation that causes the energy dissipation process. The result of this dissipation is improved fracture resistance of the liquid rubber-modified composite.

## 4. Discussion

The main causes of the clinical failure of composite fillings are secondary caries and mass fractures [22,23]. Advances in the field of the manufacture of new composite materials have enabled the introduction of synthetic liquid polybutadiene rubber, free from carcinogenic ingredients, to the market. Because of the presence of reactive vinyl groups, this liquid rubber can be incorporated into the resin polymer network, affecting its mechanical properties. Until now, research on the modification of resins with liquid rubber has focused mainly on epoxy systems [10], which are not used in dentistry. In this study, such modification was used among the first in an experimental dental composite based on dimethacrylate resins. It was possible to assess the effect of liquid rubber modification on mechanical properties and stress distribution.

The results of the mechanical tests showed that the use of liquid rubber significantly improved fracture toughness. The increase in the fracture toughness for flow material (FLR) by approximately 8% and classic composite (CLR) by approximately 12% indicates clearly that the improved fracture toughness of liquid rubber results from the presence of elastic phase in the matrix that promotes the dissipation of fracture energy. The nearly two-fold difference in fracture toughness between FLR and CLR materials is due to a different amount of reinforcement [24,25]. Additionally, the observed differences in fracture toughness may also result from different toughening mechanisms occurring in liquid rubber-modified composites, such as the formation of elastic bridges, bifurcation of the crack direction, and local plastic deformation of the matrix, which promote the dissipation of fracture energy. The effectiveness of using liquid rubber as a toughener is confirmed by the results obtained by the authors [26] in which they showed that the addition of liquid rubber led to a significant increase in the fracture toughness from 0.39 MPa·m^1/2^ to 1.24 MPa·m^1/2^ for a mixture of BisGMA/TEGDMA-based resins, confirming the effectiveness of this modification in improving mechanical properties. The effectiveness of using liquid rubber as a reinforcement agent was also confirmed in the study by Kerby et al. [27] in which it was shown that the addition of liquid rubber to BisGMA/TEGDMA resin significantly increased fracture toughness by up to 25%.

The observed increase in fracture toughness can be interpreted in the context of the fracture and strengthening mechanisms. Crack propagation is predominantly intermolecular, advancing through the matrix and along the reinforcement–matrix interface from one particle to the next. The interfacial strength is governed by the local surface area and crack pattern, resulting in particle debonding in which particles separate from the matrix yet remain attached to the opposing fracture surface. A similar cracking pattern was reported by De Souza et al. [28]. In flowable composites, fracture energy may be dissipated through several dominant mechanisms, including crack bridging, crack deflection, and the blocking of crack propagation by filler particles (Figure 7) [29,30]. After modification with liquid rubber, the propagation of the fracture changed. The rubber phase promoted the formation of elastic bridges, increasing fracture toughness and hindering crack propagation (Figure 8). These effects occur in materials with covalent bonds in networks connected by crosslinks, enabling energy dissipation and exhibiting excellent strength and elasticity [31].

Analogous to composite F, material C exhibited predominantly intermolecular cracking (Figure 9). However, the higher reinforcement volume fraction resulted in fewer crack deflections and fewer particles being bypassed by the propagating crack. The increased filler content also reduced the extent of plastic deformation in the matrix, leading to an almost complete absence of classical crack bridging and of secondary cracks formed diagonally to the primary crack path. Shah et al. [32] similarly reported the presence of bridging in microhybrid systems, noting that this mechanism transfers a portion of the load that would otherwise promote crack growth, thereby enhancing fracture toughness. Essentially, crack bridging decreases the stress concentration at the crack tip and acts as an external reinforcement mechanism. In composite C, the increased brittleness of the matrix typically favored the formation of a single dominant crack, while microcracks were sparse and generally associated with changes in crack direction. Foulk et al. [33] demonstrated that changes in crack direction during bridging provide substantially greater reinforcement than crack paths that directly traverse a reinforcement particle.

The liquid-rubber-modified CLR composite exhibited cracking mechanisms similar to those observed in composite C (Figure 10). Crack deflections accompanied by changes in propagation direction occurred primarily at larger reinforcement particles, and crack arrest or blocking was also evident at particle sites. Elastic bridging was present to a lesser extent and appeared only where the crack opening remained relatively small. The incorporation of liquid rubber is likely to facilitate plastic shearing, producing substantial crack-tip deflection and thereby enhancing energy dissipation during fracture. This increased dissipation ultimately contributes to the improved fracture toughness of the modified composite.

The increase in fracture energy imparted by the liquid rubber phase also arises from the pronounced modulus mismatch between the rigid matrix polymer and the rubber domains. Under loading, the local stress in the matrix at the scale of a rubber domain is likely to exceed the nominal applied stress. As the stress fields surrounding adjacent domains begin to overlap, the local matrix stresses intensify, leading to substantial shear stresses at the crack tip that promote plastic deformation of the matrix. Consequently, the reinforcing effect of the rubber originates from its ability to induce plastic deformation within a substantially larger volume of the matrix.

Increased fracture toughness could be associated with viscoplastic deformations of the matrix, which enable energy absorption through material deformation at the crack tip. Several liquid rubber bridges were observed, characterized by strong adhesion to the fracture edges and reinforcement particles.

These findings confirm that modification of the dental material with liquid rubber can significantly improve fracture toughness.

However, a decrease in flexural strength and Young’s modulus is observed. This is due to the introduction of an elastic phase into the structure, which reduces stiffness and stress transfer capacity. For the FLR composite, Young’s modulus decreased by ~15% and for CLR by ~16%. On the contrary, the flexural strength for the FLR composite decreased by ~14.5% and for CLR by ~11%. The results obtained are consistent with reports in the literature [34], which indicate that the presence of elastomers is responsible for a decrease in elastic modulus and flexural strength, while simultaneously increasing fracture toughness. The reduction in Young’s modulus observed in dental composites modified with liquid rubber may raise certain concerns. However, literature reports indicate that Young’s modulus values for dental composites typically range from 2.97 to 12.49 GPa [35]. The values obtained in the present study fall within this range. Moreover, ISO 4049 does not specify any requirements regarding the Young’s modulus of resin-based restorative materials.

In contrast, the incorporation of liquid rubber into dental composites led to an increase in Poisson’s ratio, indicating a change in the material’s mechanical response, particularly its deformation behavior. A higher Poisson’s ratio reflects a tendency for shape change rather than volumetric change. When subjected to tension, such materials exhibit more pronounced necking, resembling the behavior of elastomers. Under compression, materials with higher Poisson’s ratios presents a greater barreling effect, which in the context of composite restorations may influence the cavity walls.

Occlusal loads are among the primary factors influencing stress distribution within both dental tissues and restorative materials. The simulation results (Figure 5 and Figure 6) identified critical regions susceptible to potential damage, where complex loading conditions (tension and compression) may promote the formation and propagation of marginal gaps. Analysis of these results indicates that incorporating liquid rubber as a resin modifier enables a more favorable stress distribution within the restoration and along the interface with tooth tissues. In the reference material, elevated tensile stresses were observed at the enamel–restoration junction (25 MPa), which may facilitate microcrack initiation in this vulnerable region. Such stress concentrations are particularly problematic due to the brittle nature of enamel, as localized overloads may lead to chipping and increased susceptibility to mechanical failure.

Modification of the composite with liquid rubber reduced tensile stresses at the bonding interface by approximately 5 MPa, demonstrating enhanced capacity of the material to dissipate occlusal forces. Consequently, stress became more distributed within the enamel rather than concentrated in the restoration, potentially lowering the risk of composite degradation. This reduction in interfacial stress is of particular importance for the long-term stability of restorations, as it may decrease the likelihood of debonding between the filling and the tooth structure.

Modification with liquid rubber resulted in a 50% reduction in compressive stresses (Figure 6). These findings are consistent with previously published studies [27,34,36], although the present work demonstrates an even more favorable stress distribution. In the liquid-rubber-modified composite, compressive stresses were concentrated primarily along the lateral tooth walls rather than within the restoration itself, which may enhance the material’s durability and reduce the likelihood of mechanical failure. Furthermore, analysis of reduced stress distribution based on the Huber–Mises–Hencky criterion confirmed a more uniform stress pattern in the modified composite, indicating an improved capability of the material to accommodate and transfer mechanical loads. Considering the viscoelastic and partially plastic behavior introduced by liquid rubber, an even more favorable and attenuated stress distribution would be expected. However, at this stage of the research, our intention was to examine the stress behavior specifically at the restoration surface as its material properties were altered.

## 5. Conclusions

The present study demonstrates that the incorporation of liquid rubber into dental composites significantly modifies their mechanical behavior and fracture response. Incorporation of liquid rubber increased fracture toughness by 8–12% in experimental dental composites via crack bridging, deflection, and plastic deformation mechanisms. Modified composites exhibit increased crack-tip deflection and elastic bridging, reducing propagation of cracks compared with unmodified materials. Finite element analysis showed reduced tensile (≈5 MPa) and compressive (≈50%) stresses, with stress concentrated along lateral tooth walls rather than the restoration, which may promote durability and lowering failure risk. Young’s modulus and flexural strength decreased (11–16%), while Poisson’s ratio increased, reflecting improved deformation behavior without compromising clinically acceptable performance. Improved fracture toughness and stress distribution suggest greater long-term restoration stability, reduced marginal gaps, and lower risk of mechanical failure under occlusal loads.

Based on the experimental and numerical results, the null hypothesis can be considered confirmed. Liquid rubber modification led to improved stress distribution and reduced susceptibility to crack initiation and propagation, ultimately enhancing the fracture toughness and performance under occlusal loads.

## Figures and Tables

**Table 1:** Designation of tested materials.

Material	Mark
Flow type composite	F
Flow-type composite modified with 5% liquid rubber	FLR
Classic composite	C
Classic composite modified with 5% liquid rubber	CLR

**Table 2:** The properties of the tooth tissue adopted for FEA analysis based on work [19].

Details	Enamel	Dentin
Young’s modulus [GPa]	84.10	18.60
Poisson’s ratio	0.33	0.31
Density [g/cm^3^]	2.90	4.0

**Table 3:** Experimental properties of composites adopted for FEA analysis.

Details	F	FLR	C	CLR
Fracture toughness [MPa·m^1/2^]	1.04	1.13	1.97	2.18
Flexural strength [MPa]	71.90	61.48	102.30	90.96
Young’s modulus [GPa]	2.98	2.53	7.33	6.16
Poisson’s ratio	0.24	0.29	0.32	0.39

## Data Availability

The original contributions presented in this study are included in the article. Further inquiries can be directed to the corresponding author.
